# Honey reduces blood alcohol concentration but not affects the level of serum MDA and GSH-Px activity in intoxicated male mice models

**DOI:** 10.1186/s12906-015-0766-5

**Published:** 2015-07-14

**Authors:** Peiying Shi, Bing Chen, Conghai Chen, Jingyang Xu, Zhenhuang Shen, Xiaoqing Miao, Hong Yao

**Affiliations:** Department of Traditional Chinese Medicine Resource and Bee products, College of Bee Science, Fujian Agriculture and Forestry University, Fuzhou, 350002 China; Department of Pharmaceutical Analysis, Faculty of Pharmacy, Fujian Medical University, Fuzhou, 350004 China; State and Local Joint Engineering Laboratory of Natural Biotoxins, Fujian Agriculture and Forestry University, Fuzhou, 350002 China

## Abstract

**Background:**

For a long time, honey was purportedly helpful to prevent drunkenness and relieve hangover symptoms. However, few of the assertions have experienced scientific assessment. The present study examined the effects of honey on intoxicated male mice.

**Methods:**

Low or high doses of lychee flower honey (2.19 or 4.39 g/kg body weight, respectively) were single orally administrated 30 min before the ethanol intoxication of mice, followed by recording the locomotor activity by autonomic activity instrument and observing the climbing ability after alcohol. On the other hand, 2.19 g/kg honey was single orally administrated 5 min after the ethanol intoxication of mice, followed by determining the ethanol concentration in mice blood. In addition, subacute alcoholism mice models were developed and after the treatment of 2.19 g/kg honey s.i.d for successive three days, the level of serum malondialdehyde (MDA) and glutathione peroxidase (GSH-Px) activity were detected in the models.

**Results:**

Both of the two doses of honey increased the autonomic activity of alcoholized mice. Furthermore, the treatment of 2.19 g/kg honey could decrease significantly the blood ethanol concentration in intoxicated mice. The anti-intoxication activity of honey could be due to the effect of the fructose contained in the honey. Meanwhile, honey could not affect the serum MDA level and GSH-Px activity in alcoholism mice models.

**Conclusion:**

Honey indeed possesses anti-intoxication activity.

## Background

Natural honey is one remarkable liquid bee product, which is rich in fructose and glucose and contains appreciable amount of disaccharides, trisaccharides, minerals, vitamins, amino acids, proteins and some organic acids, *etc.* [1]. From ancient to nowadays, honey is always an important pharmaceutical and food resource all over the world. In the Shennong's Materia Medica Classic (Shennong Bencao Jing), an ancient traditional Chinese medicine record, it describes that “honey could settle internal organs, invigorate spleen and replenish qi, relieve pain and detoxify, heal over hundreds of diseases, make hundreds of drugs harmonized and prolong life by long-term use”. Especially, for a long time, honey was purportedly used to prevent drunkenness and relieve hangover symptoms in folk in China. Modern pharmacological investigations confirmed the multiple function effects of honey referring to antimicrobial activity [2], gastric protection [3–8], improving blood profiles [9], cario-protection [10], and ameliorating risk factors of metabolic and cardiovascular diseases [1], *etc*. The anti-intoxication effects of honey were also involved indirectly, such as preventing ethanol-induced gastric lesions in rats [6, 7]. Nevertheless, up to nowadays, few of the assertions for honey anti-intoxication have obtained supports from direct scientific assessment.

The present study aims to evaluate the anti-intoxication effects of honey on alcoholized male mice. Firstly, the intoxication dosage was optimized to establish intoxication animal models in male mice. With the pretreatment of lychee flower honey 30 min before intoxication, the autonomic activity in 60 min and climbing ability of alcoholized mice at 65 min were recorded. Meanwhile, with the treatment of honey at 5 min after administration of ethanol, the ethanol concentrations in blood of the alcoholized mice were determined by gas chromatography at 30 min after administration of honey. In addition, the levels of serum malondialdehyde (MDA) and glutathione peroxidase (GSH-Px) activity in subacute alcoholism mice models were determined. Finally, the results are discussed as referring to the anti-intoxication effect and the possible effect mechanism of honey.

## Methods

### Regents and drugs

Saline (0.9 % NaCl, No. 121112B43) was purchased from Fuzhou Neptunus Futao Pharmaceuticals Co. Ltd., China. Alcohol (Sinopharm Chemical Reagent Co., Ltd., Shanghai, China) was diluted with saline (0.9 % NaCl) to 53 % v/v for this study. Honey of lychee flower (FM020204130301) was provided by Fujian Shenfeng Science Development Co. Ltd., China. Positive drug, “RU-21 wake up clear” pills (lot#25727, Spirit Sciences, USA), a commercial dietary supplement for consumers of alcohol, was purchased from local market. For serum MDA and GSH-Px assay, MDA and GSH-Px kits were purchased from Nanjing Jiancheng Bioengineering Institute, China.

### Apparatus

YLS-1A type mouse autonomic activity recorders (Jinan Yiyan Science and Technology Development Co. Ltd.) were used in locomotor activity experiments.

For blood ethanol analysis, GC-2010 gas chromatography (Shimadzu, Japan) was used with DB-wax column (30 m × 250 μm × 0.25 μm) and hydrogen flame ion detector. The injector temperature was set at 150 °C. The temperatures of column and detector were set at 55 °C and 200 °C, respectively.

### Animals

Adult male Kunming mice (8 to 10 weeks old and 20 to 30 g body weight; Shanghai Slac Laboratory Animal Co. Ltd., Shanghai, China) were used extensively. All mice were grouphoused and maintained at a 12/12 hour light/dark cycle. The environment temperature was maintained at 25 °C with 50 % humidity and tap water and food were supplied ad libitum to the animals. All animal manipulations were performed under official license from the experimental animal ethics committee of Fujian Medical University, China.

### Drunken dosage optimization

40 mice were classified randomly into 4 groups (10 mice per group). One group mice were orally administrated 0.15 ml/10 g body weight (BW) saline. The other groups’ mice were orally administrated single dose of 0.05, 0.10 or 0.15 ml/10 g BW 53 % (v/v) ethanol. The locomotor activity was recorded in 30 min as described in the next locomotor activity experiments. Subsequently, the climbing behavior of the studied mice was observed as described in the next climbing ability test at 30 ~ 40 min.

### Locomotor activity experiments

Locomotor activity was recorded as described previously [11, 12]. Briefly, mice were placed into sound-attenuated, ventilated, and dim lit locomotor boxes. Photocell detection allowed a computer-based system to register the activity of the mice. Locomotor activity was defined as the accumulated number of photocell beams interrupted during a 30 or 60 minute period. The mice were allowed to habituate to the locomotor activity box 1 hour prior to drug administration.

### Climbing ability test

For further identifying the effect of drug on locomotor behavior, mice were placed onto the metal grid lids of mouse cages. The lids were then placed vertically to observe the climbing time of studied mice at the lids, which were assessed as the climbing ability and could reflect the effect of drug on locomotor behavior of mice.

### Drug administration

For investigating the prevention of honey from getting drunk, 60 male mice were randomly classified into six groups (10 mice per group), including low dose of honey group (T1 group, pretreated by 2.19 g/kg BW dose honey at 30 min before alcohol, diluting into 0.2 ml with saline), high dose of honey group (T2 group, pretreated by 4.39 g/kg BW dose honey at 30 min before alcohol, diluting into 0.2 ml with saline), negative control group (T0 group, pretreated by 0.2 ml saline at 30 min before alcohol), positive control group (P group, pretreated by 0.195 g/kg BW dose RU-21 30 min before alcohol, suspending with 0.2 ml saline), normal group (C group, pretreated by 0.2 ml saline and without administration of alcohol), and normal control group (N group, pretreated by 2.19 g/kg BW dose honey and without administration of alcohol). Before test, all the mice were placed into the YLS-1A recorders to habituate to the locomotor activity box 1 hour. Subsequently, each group mice were orally administrated the optimum single dose of 53 % v/v ethanol at 30 min after pretreated by honey, RU-21, or saline except for normal group mice and were placed back into the locomotor activity box. After administration of alcohol, the locomoter activity of each mouse was registered by the recorder within 60 min.

For studying the healing effect of honey on intoxicated animal models, 20 male mice were randomly classified into two groups (10 mice per group), including treatment group (treated with 2.19 g/kg BW dose honey at 5 min after alcohol, diluting into 0.2 ml with saline) and model group (treated with 0.2 ml saline at 5 min after alcohol). Before test, all the mice were placed in mice cage to habituate to the environment for 1 day. The climbing ability of all mice was assessed at 30 min after drug administration. About 0.5 ml of whole blood was then collected from each group of mice via enucleation of eyeball at about 35 min after alcohol. The whole blood was used to analyze the blood ethanol concentration.

### Blood ethanol concentration determination

Blood ethanol concentration was determined using headspace capillary gas chromatography (GC) according to previous reports [13] with slight modification. In brief, for preparation of calibration curve, 0.1 ml of ethanol standard solution (0.5, 1, 3, 5, 7, or 9 mg/ml in water) and 0.5 ml internal solution (100 μg/ml tert-butyl alcohol in water) were transferred into headspace sample bottles (10 ml volume) and vortexed for at least 30 s, followed by screwing the lids. For sample analysis, 0.1 ml of whole blood instead of the ethanol standard solution was transferred into the bottle and the next procedures were completely same to those in the preparation of calibration curve. All the bottles were then heated with 50 °C waterbath for 30 min. 100 μl of headspace air was injected into the GC for analysis.

### Serum MDA and GSH-Px in subacute mice models of alcoholism

18 mice were randomly classified into three groups (6 per group), including negative group, model group, and treatment group. All mice were allowed to hibitute to the cagehouse for 3 days. The mice in model group and treatment group were orally administrated 0.1 ml/20 g BW dose 53 % v/v ethanol, followed by oral administration of 0.2 ml saline and 0.2 ml honey solution (2.19 g/kg BW dose honey) at about 5 min after ethanol, respectively. The negative group mice were not administrated ethanol but treated with 0.2 ml saline. The intoxication and treatment were performed successively for 3 days. About 0.5 ml whole blood were collected from each group of mice via enucleation of eyeball at about 30 min after the last administration. The blood was placed in a 4 °C refrigerator for 30 min, followed by centrifugation with 3000 rpm to obtain about 0.2 ml serum. All the serum was stored at −20 °C before test. The determination of serum MDA and GSH-Px were performed using MDA and GSH-Px kits according to the kits procedure introduction.

### Statistical analysis

All data analysis was performed with an unpaired-test (*t*-test). Data are presented as mean ± SD. A probability value of p < 0.05 was considered as statistically significant.

## Results

### Optimization of intoxication dosage for mice

The locomotor activity and climbing behavior of studied mice were recorded in 30 min in the model establishment trial. As listed in Table 1, the locomotor activity and climbing time of the alcoholized mice are decreased with the increasing of alcohol dosage. Especially, a statistically significant effect of alcohol on locomotor activity and climbing time were observed for group 3 and 4 (*vs.* group 1, p < 0.05). However, three mice in group 4 were comatose at 30 min after alcohol. Obviously, the dosages, both of 0.1 ml/10 g and 0.15 ml/10 g dose could intoxicate the mice, but 0.15 ml/10 g dose could be close to the fatal dose for mice. In the next studies, the dose of 0.1 ml/10 g dose was selected for preparing drunken mice models.

**Table 1:** The optimization results of intoxication dosage for mice (mean ± SD, n = 10)

Groups	Dosage/10 g body weight	Locomotor activity numbers	Climbing time (S)
1	0.15 ml saline	657.9 ± 142.1	882.7 ± 211.4
2	0.05 ml alcohol	526.7 ± 121.1	851.3 ± 612.1
3	0.1 ml alcohol	246.3 ± 227.8*	441.4 ± 175.8*
4	0.15 ml alcohol	177.9 ± 161.5*	10.9 ± 12.3*

*p < 0.05, *vs*. group 1

### Prevention drunkenness

**Figure 1:**
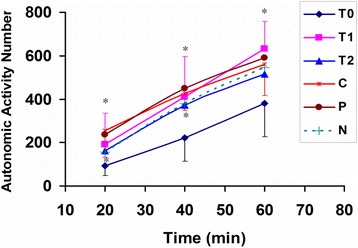
The prevention of honey from drunkenness in mice. *T0 group*, pretreated by saline 30 min before ethanol; *T1 group*, pretreated by 2.19 g/kg body weight dose honey at 30 min before alcohol (*p < 0.05, *vs.* T0, *n* = 10 per group); *T2 group*, pretreated by 4.39 g/kg body weight dose honey at 30 min before alcohol; *P group*, pretreated by 0.195 g /kg body weight dose RU-21 at 30 min before alcohol; *C group*, pretreated by saline and without administration of alcohol (* *p* < 0.05, *vs.* T0); *N group*, pretreated by 2.19 g/kg body weight dose honey and without administration of alcohol. The data for T0, T1 and C groups represent mean ± SD, and the others represent mean

As shown in Fig. 1, the locomotor activity of model group (T0 group) is significantly decreased compared with that of normal group (C group) at 40 min after alcohol. It demonstrated that the drunken animal model indeed succeeded. A statistically significant overall effect of low dose honey pretreatment for T1 group (2.19 g/kg BW dose honey) was observed (*p* < 0.05, *vs.* T0 group, *n* = 10 per group) within 60 min after alcohol. The effect was also observed for T2 group (4.39 g/kg BW dose honey) at 20 min and 40 min (*p* < 0.05, *vs*. T0 group, *n* = 10 per group). In addition, the statistically significant effect could be also observed for P group (positive drug, RU-21) at 20 min and 40 min (*p* = 0.03 and 0.07, respectively, *vs.* T0 group, *n* = 10 per group) after ethanol. There were no significant differences between N and C groups.

### Effect of honey on blood ethanol concentration in alcoholized mice

The ethanol determination results were listed in Table 2. As shown in Table 2, the ethanol concentration for treatment group (treated with 2.19 g/kg BW dose honey 5 min after alcohol) has statistically significant difference from that for model group (treated with saline after alcohol). The treatment of 2.19 g/kg BW dose honey made the ethanol concentration decreased about 36 % compared to the model group at 30 min after treatment. Meanwhile, there is statistically significant difference between the treatment group and model group for climbing time, suggesting that the honey treatment also made the autonomic activity increased.

**Table 2:** The results of the climbing test and ethanol content in mice blood (Mean ± SD, n = 10)

Groups	Administration	Blood ethanol concentration (mg/ml)	Climbing time (s)
Model group	0.2 ml saline	4.99 ± 1.16	419.1 ± 353.8
Treatment group	0.2 ml, 2.19 g/kg body weight dose honey	3.19 ± 0.81*	672.5 ± 298.7*

Note: ^*^ *p* < 0.05, *vs.* model group

### Effect of honey on serum MDA level and GSH-Px activity in alcoholism mice models

As shown in Table 3, there is significant difference between model group and negative group for MDA and GSH-Px values, suggesting that the successive administration of ethanol induced the change of MDA level and GSH-Px activity in mice serum and the subacute alcoholism mice models should be successfully established. However, there was no significant difference observed between the model group and the treatment group for MDA and GSH-Px values.

**Table 3:** The results of MDA and GSH-Px in mice blood (Mean ± SD, n = 6)

Groups	Administration	MDA (nmol/ml)	GSH-Px (U)
Negative	0.2 ml saline	5.46 ± 1.35	965.51 ± 131.71
Model	0.2 ml saline	6.61 ± 1.80*	234.26 ± 95.69*
Treatment	0.2 ml, 2.19 g/kg body weight dose honey	6.58 ± 3.04	250.79 ± 117.45*

Note: ^*^ *p* < 0.05, *vs.* negative group

## Discussion

**Figure 2:**
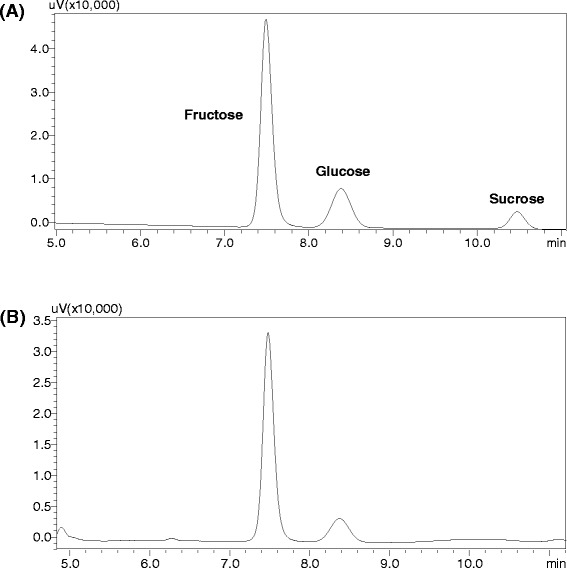
The HPLC chromatograms of (**a**) mixture standard solutions including 4 mg/ml fructose, 3 mg/ml glucose and 1 mg/ml sucrose, and (**b**) honey samples (9.4 mg/ml). Conditions: High-performance liquid chromatographic analysis was carried out using a Shimadzu 20A series HPLC system (Japan) equipped with two pumps with column oven and ultraviolet detector; a Welch ultimate XB-NH_2_ column (4.6 mm × 250 mm, 5 μm) was used, sample injection volume was 20 μl, the temperature of column oven was set at 30 °C, flow rate was 1 ml/min, a isocratic elution was performed with mobile phases consisted of water and acetonitrile (30 : 70) and the monitored wavelength was set at 190 nm

The results from the pretreatment of lychee flower honey showed that there is apparent preventing drunkenness effect in mice model for low dose of honey (2.19 g/kg BW) within 60 min after administration of alcohol. The prevention was also observed in mice for high dose of honey (4.39 g/kg BW) at 20 min and 40 min after alcohol. The results hinted that honey could be indeed helpful to prevent drunkenness. Since the major components contained in the studied honey are fructose (38.06 %, g/g) and glucose (32.67 %, g/g), which had been determined by a HPLC method (Fig. 2), it is reasonable to presume that the fructose or/and glucose resulted in the preventing drunkenness effect in alcoholized mice. The preventing effect in mice could be due to that fructose and/or glucose inhibited the absorption of ethanol in mice gastrointestinal tract or enhanced the elimination of ethanol in intoxicated mice. This hypothesis could obtain positive support from the observations that adult female rats, fed chow diets supplemented with fructose or glucose in their drinking water for 10 days demonstrated significantly greater ethanol elimination rates (4.85 ± 0.28 and 4.92 ± 1.5 μM ethanol/min/g liver, respectively) than rats receiving water (3.65 ± 0.29) [14]. In fact, it was also observed that the treatment of honey in alcoholized male mice could significantly reduce the blood alcohol concentration at 30 min after administration of honey in the present study. However, in the early clinical study, fructose infusions resulted in a lowering of the alcohol levels in the blood of man by 43 % compared to the values obtained during saline solution infusions and glucose infusions were ineffective [15]. It demonstrated that glucose could have no significant effect on the rate of ethanol elimination in men. A literature also reported that treatment with glucose was ineffective in reducing blood alcohol level in male rats with acute ethanol intoxication [16]. These reported results suggest that the subjects’ sex or genus could exert indeterminacy influence on honey (being rich in carbohydrates fructose and glucose) enhancing alcohol elimination or reducing blood alcohol concentration.

An early clinical investigation demonstrated that neither fructose nor glucose had any significant effect on the intensity of alcohol intoxication and hangover [17]. However, glucose and fructose significantly inhibited some metabolic alterations (increasing blood lactate, free fatty acid and ketone body *etc.* in hangover male volunteers) induced by ethanol in these subjects and in this respect fructose was more effective than glucose [17]. Meanwhile, high fructose intake could also be associated with adverse metabolic syndromes [18], which could be a rational explanation to the results that low dose of honey showed the prevention better than high dose of honey in alcoholized mice. Although dietary carbohydrate supplementation (fructose or glucose) enhanced ethanol elimination, it did not alter the activity of alcohol dehydrogenase, which suggested that the changes in the ethanol elimination rate following carbohydrate loading were not the consequence of an alteration in hepatic alcohol dehydrogenase [14]. Ethanol-induced liver injury associated with increased oxidative stress and free radical-mediated tissue damage was widely demonstrated in rats and humans [19]. Free radicals or reactive oxygen species are responsible for ethanol induced oxidative stress [19]. Free radicals formed from the ethanol mediated process have a great potential to react rapidly with lipids, which in turn leads to lipid peroxidation (LPO). The level of MDA has been widely used as a biomarker of LPO for many years [19]. Accordingly, the level of MDA might also be considered as an alcohol-induced liver injury index. In our study, the significant increase in serum MDA concentration in alcoholism mice models was observed. There was no significant difference in serum MDA concentration between honey treatment group and model group, which were accordance with the previous reports that the consumption of fructose did not facilitate significant changes in rat plasma MDA level [20]. Meanwhile, free radical scavenging enzymes such as GSH-Px, SOD and GST *etc.* are the first line of defense against oxidative injury [19, 21, 22]. Alcohol-induced oxidative stress could deplete the antioxidant enzyme system, resulting in the extremely decreased enzyme activity. Indeed, the GSH-Px activity of alcoholism model group was significantly lowered than that of negative group and the honey treatment had no significant positive effect on the alcohol-induced GSH-Px activity descent. The results were accordance with the previous reports that feeding the fructose diets decreased the activity of GSH-Px by 25 and 50 % in the copper-supplemented and copper-deficient rats, respectively [23].

In addition, besides the main components carbohydrates, honey contains essential elements (calcium, phosphorus, iron, sodium, potassium), as well as compounds like proteins and vitamins [24]. All these substances give the nutritional properties of honey [24]. The special odour and taste of honey make people be willing to enjoy it. Furthermore, honey has been particularly recommended for children and sportsmen, because it helps improving the organism efficiency (children, elderly and invalids) [24]. Therefore, although possessing anti-drunkenness property [25], fructose is not able to replace honey to be used due to the preeminent heath care properties of honey.

## Conclusions

In summary, honey indeed could reduce blood alcohol concentration, which could mainly result from the effect of the fructose contained in the honey. In addition, the results of MDA level and GSH-Px activity suggested honey could not be a promising food supplement against alcoholic liver injury in mice.

## Abbreviations

GC: gas chromatography
GSH-Px: glutathione peroxidase
LPO: lipid peroxidation
MDA: malondialdehyde.

## References

[1] Ajibola A, Chamunorwa JP, Erlwanger KH (2012). Nutraceutical values of natural honey and its contribution to human health and wealth. Nutr Metab.

[2] Alvarez-Suarez JM, Tulipani S, Diaz D, Estevez Y, Romandini S, Giampieri F, Damiani E, Astolfi P, Bompadre S, Battino M (2010). Antioxidant and antimicrobial capacity of several monofloral Cuban honeys and their correlation with color, polyphenol content and other chemical compounds. Food Chem Toxicol.

[3] Ali AT (1995). Natural honey exerts its protective effects against ethanol-induced gastric lesions in rats by preventing depletion of glandular non protein sulfhydryls. Trop Gastroenterol.

[4] Ali AT, al-Swayeh OA, al-Humayyd MS, Mustafa AA, al-Rashed RS, Al-Tuwaijiri AS (1997). Natural honey prevents ischaemia-reperfusion-induced gastric mucosal lesions and increased vascular permeability in rats. Eur J Gastroenterol Hepatol.

[5] al-Swayeh OA, Ali AT (1998). Effect of ablation of capsaicin sensitive neurons on gastric protection by honey and sucralfate. Hepatogastroenterology.

[6] Gharzouli K, Gharzouli A, Amira S, Khennouf S (1999). Prevention of ethanol-induced gastric lesions in rats by natural honey and glucose-fructose-sucrose-maltose mixture. Pharmacol Res.

[7] Gharzouli K, Amira S, Gharzouli A, Khennouf S (2002). Gastro protective effects of honey and glucose-fructose-sucrose-maltose mixture against ethanol-, indomethacin-, and acidified aspirin induced lesions in the rat. Exp Toxicol Pathol.

[8] Nasuti C, Gabbianelli R, Falcioni G, Cantalamessa F (2006). Antioxidative and gastro protective activities of anti-inflammatory formulations derived from chestnut honey in rats. Nutr Res.

[9] Chepulis LM (2007). The effects of honey compared with sucrose and a sugar-free diet on neutrophil phagocytosis and lymphocyte numbers after long-term feeding in rats. J Complement Integr Med.

[10] Molan P (1999). Why honey is effective as a medicine. 1. Its use in modern medicine. Bee World.

[11] Jerlhag E, Egecioglu E, Dickson SL, Andersson M, Svensson L, Engel JA (2006). Ghrelin stimulates locomotor activity and accumbal dopamine-overflow via central cholinergic systems in mice: implications for its involvement in brain reward. Addict Biol.

[12] Jerlhag E, Ivanoff L, Vater A, Engel JA (2014). Peripherally circulating ghrelin does not mediate alcohol-induced reward and alcohol intake in rodents. Alcohol Clin Exp Res.

[13] Colombo G, Agabio R, Lobina C, Reali R, Morazzoni P, Bombardelli E, Gessa GL (1999). *Salvia miltiorrhiza* extract inhibits alcohol absorption, preference, and discrimination in sP rats. Alcohol.

[14] Keegan A, Batey R (1993). Dietary carbohydrate accelerates ethanol elimination, but does not alter hepatic alcohol dehydrogenase. Alcohol Clin Exp Res.

[15] Lowenstein LM, Simone R, Boulter P, Nathan P (1970). Effect of Fructose on Alcohol Concentrations in the Blood in Man. JAMA.

[16] Portari GV, Marchini JS, Vannucchi H, Jordao AA (2008). Antioxidant effect of thiamine on acutely alcoholized rats and lack of efficacy using thiamine or glucose to reduce blood alcohol content. Basic Clin Pharmacol Toxicol.

[17] Ylikahri RH, Leino T, Huttunen MO, Pösoö AR, Eriksson CJP, Nikkilä EA (1976). Effects of fructose and glucose on ethanol-induced metabolic changes and on the intensity of alcohol intoxication and hangover. Eur J Clin Invest.

[18] Zhao Y, Yang XB, Ren DY, Wang DY, Xuan Y (2014). Preventive effects of jujube polysaccharides on fructose-induced insulin resistance and dyslipidemia in mice. Food Funct.

[19] Hou ZH, Qin PY, Ren GX (2010). Effect of anthocyanin-rich extract from black rice (*Oryza sativa* L. *Japonica*) on chronically alcohol-induced liver damage in rats. J Agric Food Chem.

[20] Zagrodzki P, Joniec A, Gawlik M, Gawlik M, Krośniak M, Fołta M, Bartoń H, Paśko P, Chłopicka J, Zachwieja Z (2007). High fructose model of oxidative stress and metabolic disturbances in rats. Part I. Antioxidant status of rats’ tissues. Bull Vet Inst Pulawy.

[21] Chiang AN, Wu HL, Yeh HI, Chu CS, Lin HC, Lee WC (2006). Antioxidant effects of black rice extract through the induction of superoxide dismutase and catalase activities. Lipids.

[22] Halliwell B (1994). Free radicals, antioxidants and human diseases curiosity, causes or consequence. Lancet.

[23] Fields M, Ferretti RJ, Cecil Smith J, Reiser S (1984). Interaction between dietary carbohydrate and copper nutriture on lipid peroxidation in rat tissues. Biol Trace Elem Res.

[24] Dobrinas S, Soceanu A, Matei N, Danciu I (2010). Validation of a spectrometric method based on prussian blue reaction used for the determination of ascorbic acid from honey and propolis. Ovidius University Annals of Chemistry.

[25] Onyesom I, Anosike EO (2004). Oral fructose-induced changes in blood ethanol oxidokinetic data among healthy nigerians. Southeast Asian J Trop Med Public Health.

